# Fecal Microbiota Underlying the Coexistence of Schizophrenia and Multiple Sclerosis in Chinese Patients

**DOI:** 10.1155/2023/5602401

**Published:** 2023-08-30

**Authors:** Li Shao, Jinlong Fu, Lulu Xie, Guangyong Cai, Yiwen Cheng, Nengneng Zheng, Ping Zeng, Xiumei Yan, Zongxin Ling, Shiwei Ye

**Affiliations:** ^1^School of Clinical Medicine, Institute of Hepatology and Metabolic Diseases, Hangzhou Normal University, The Affiliated Hospital of Hangzhou Normal University, Hangzhou, Zhejiang, China; ^2^Rugao Experimental Primary School, Nantong, China; ^3^Department of Rehabilitation Medicine, Lishui Second People's Hospital, Lishui, China; ^4^Collaborative Innovation Center for Diagnosis and Treatment of Infectious Diseases, State Key Laboratory for Diagnosis and Treatment of Infectious Diseases, National Clinical Research Center for Infectious Diseases, The First Affiliated Hospital, School of Medicine, Zhejiang University, Hangzhou, Zhejiang, China; ^5^Jinan Microecological Biomedicine Shandong Laboratory, Jinan, China; ^6^Department of Obstetrics, The First Affiliated Hospital, School of Medicine, Zhejiang University, Hangzhou, Zhejiang, China; ^7^Department of Psychiatry, Lishui Second People's Hospital, Lishui, China

## Abstract

Both schizophrenia (SZ) and multiple sclerosis (MS) affect millions of people worldwide and impose a great burden on society. Recent studies indicated that MS elevated the risk of SZ and vice versa, whereas the underlying pathological mechanisms are still obscure. Considering that fecal microbiota played a vital role in regulating brain functions, the fecal microbiota and serum cytokines from 90 SZ patients and 71 age-, gender-, and BMI-matched cognitively normal subjects (referred as SZC), 22 MS patients and 33 age-, gender-, and BMI-matched healthy subjects (referred as MSC) were analyzed. We found that both diseases demonstrated similar microbial diversity and shared three differential genera, including the down-regulated *Faecalibacterium*, *Roseburia*, and the up-regulated *Streptococcus*. Functional analysis indicated that the three genera were involved in pathways such as “carbohydrate metabolism” and “amino acid metabolism.” Moreover, the variation patterns of serum cytokines associated with MS and SZ patients were a bit different. Among the six cytokines perturbed in both diseases, TNF-*α* increased, while IL-8 and MIP-1*α* decreased in both diseases. IL-1ra, PDGF-bb, and RANTES were downregulated in MS patients but upregulated in SZ patients. Association analyses showed that *Faecalibacterium* demonstrated extensive correlations with cytokines in both diseases. Most notably, *Faecalibacterium* correlated negatively with TNF-*α*. In other words, fecal microbiota such as *Faecalibacterium* may contribute to the coexistence of MS and SZ by regulating serum cytokines. Our study revealed the potential roles of fecal microbiota in linking MS and SZ, which paves the way for developing gut microbiota-targeted therapies that can manage two diseases with a single treat.

## 1. Introduction

Schizophrenia (SZ), characterized by positive and negative symptoms such as delusions, hallucinations, apathy, thought disorder, apathy, and cognitive and functional impairment, is a complex psychiatric neurodevelopmental disorder, affecting more than 20 million people worldwide and over 7 million people in China [1]. Multiple sclerosis (MS), which is associated with myelin loss, varying degrees of axonal pathology, and progressive neurological dysfunction, is a multifactorial disease of the central nervous system (CNS), affecting nearly 2.3 million people worldwide with a prevalence of 50-300/100,000 [2]. Both SZ and MS are leading causes of disability worldwide and impose heavy psychosocial burden for caregivers and high costs on society. Recently, some studies have suggested a link between MS and SZ [3]. It was proposed that the immune dysregulation that affects CNS function in MS may also play a role in SZ [4]. There was also a significantly elevated risk of SZ in prior-recorded MS and of subsequent MS in people with prior-recorded SZ [5]. Moreover, MS and SZ patients show varying degrees of dysfunctions in immune and brain, suggesting possible shared mechanisms [6, 7]. However, the pathological mechanisms underlying the co-occurring of both diseases remain elusive.

Most recently, Ahangari et al. reanalyzed the largest available genome-wide association analysis (GWAS) datasets for SZ and MS and identified the shared genetic architecture of both diseases [8]. Other than the effects of genetic and antigenic exposures [9], gut microbiota have been found to play a vital role in various brain diseases such as neurodegenerative disease via the gut microbiome-brain axis [10]. The potential roles of gut microbiota in modulating the immune system and the homeostasis of the central nervous system can be mediated by multiple kinds of microbiota-related molecules, such as cytokines, metabolites, and even bacterial extracellular vesicles [11–13]. Mounting evidences suggested that patients with MS from western developed countries and China as well as patients with various stages of SZ demonstrated gut microbial dysbiosis with both depletion and enrichment of certain bacteria as compared to controls. Jangi et al. reported an increase of *Methanobrevibacter*, *Akkermansia*, and decrease of *Prevotella*, *Faecalibacterium* in patients with MS as compared to controls [14]. We found that several key functional bacteria, primarily *Faecalibacterium*, decreased remarkably in stable Chines MS patients [15]. We also reported that Chinese elderly SZ patients were associated with decreased abundances of *Faecalibacterium*, *Roseburia*, and increased abundances of *Prevotella*, *Akkermansia* [16]. Moreover, transplantation of gut microbiota from drug-free SZ patients led to SZ-like behavioral abnormalities and dysregulated kynurenine metabolism in mice [17]. In addition,, both SZ and MS patients demonstrated significantly perturbed profiles of proinflammatory and anti-inflammatory cytokines and chemokines, which demonstrated significant correlations with certain fecal microbiota in patients [15, 16]. Such results indicated that fecal microbiota dysbiosis participated in the development of both diseases and may contribute to the associations between SZ and MS.

Considering the findings that the microbiome influences on neuroimmune interactions in neurodegenerative disease [18, 19], we reanalyzed the fecal microbial sequencing data and serum cytokines from Chinese patients with SZ and MS published in our previous studies [15, 16], with the aim to probe the microbial disorders that link the development of both diseases. Our study may pave the way for developing gut microbiota-targeted intervention strategies capable of treating one disease and reducing the incidence of the other disease at the same time.

## 2. Methods

### 2.1. Datasets

Datasets deposited in the Sequence Read Archive with accession numbers PRJNA807473 and SRP258890 were retrieved and reanalyzed in this study [15, 16]. Both datasets contained raw reads of fecal microbiota targeting the V3-V4 regions of rRNA gene sequenced with Illumina MiSeq instrument [20]. Dataset PRJNA807473 was obtained from 90 well-controlled Chinese elderly SZ patients and 71 age-, gender-, and BMI-matched cognitively normal subjects (referred as SZC), while dataset SRP258890 was from 22 patients with MS and 33 age-, gender-, and BMI-matched healthy subjects (referred as MSC). All SZ patients were diagnosed according to the criteria of the Diagnostic and Statistical Manual of Mental Disorders Fourth Edition (DSM-IV), and they were not treated with any psychiatric drugs such as antidepressants and mood stabilizers during the last 1 month. SZ patients meeting either of the following criteria were excluded: body mass index higher than 28 kg/m^2^; family history of dementia; any kind of other neurodegenerative diseases such as Alzheimer's disease or Parkinson's disease; any kind of mental disease such as depression; any kind of tumor; treated with psychiatric drugs such as antidepressants, mood stabilizers, and so on; known active infections such as bacterial, viral, or fungal infection; other diseases such as irritable bowel syndrome, inflammatory bowel disease, or other autoimmune diseases. MS patients, diagnosed based on the 2005 McDonald criteria, were stable and not of new onset or active relapse, and they did not receive immunosuppressive medications such as steroids and beta-interferon/glatiramer acetate in the preceding 3 months. MS patients meeting either of the following criteria were excluded: less than 20 years old; body mass index higher than 30 kg/m^2^; pregnancy; known active infections such as bacterial, viral, or fungal infections; use of antibiotics, probiotics, or prebiotics in the last month; any other diseases such as hypertension, diabetes mellitus, inflammatory bowel disease, irritable bowel syndrome, and other autoimmune diseases. The levels of cytokines quantified with the Bio-Plex assay (Bio-Rad) in serum samples of corresponding patients (MS, SZ) and controls (MSC, SZC) were also retrieved. Detailed information about the samples and datasets was provided in our previous studies [15, 16].

### 2.2. Study Design

**Figure 1:**
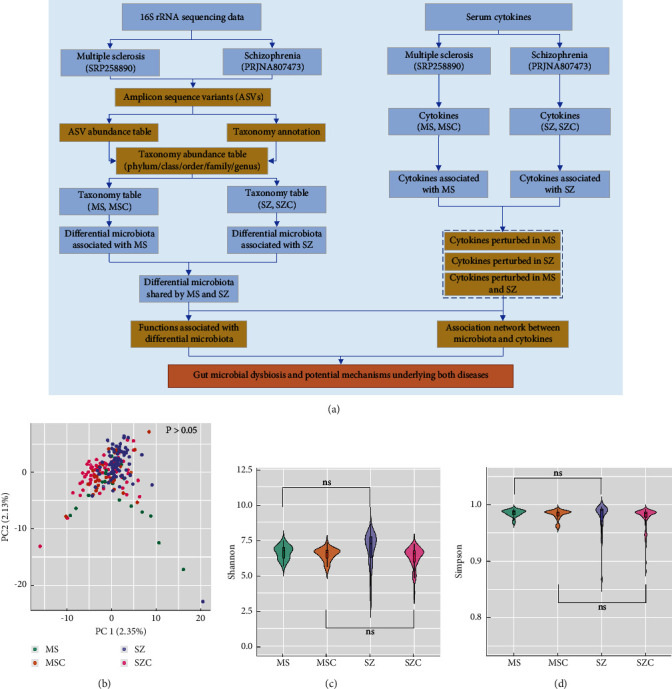
(a) A schema of the study design. (b) PCA score plot of ASV abundance tables. (c) Shannon index for MS patients, SZ patients, and corresponding controls. (d) Simpson index for MS patients, SZ patients, and corresponding controls.

Figure 1(a) demonstrated a schema of the study design. After retrieving the sequencing reads from both datasets, the reads from SZ and MS patients as well as corresponding controls (SZC and MSC) were processed with the same pipeline and parameters and pooled together for ASV (amplicon sequence variants) clustering, taxonomy annotation, and quantification. Based on the abundance table of microbiota obtained from the results of ASV quantification and taxonomy annotation, microbiota differential between MS patients and MSC, SZ patients and SZC were retrieved, separately. At the same time, the cytokines differed between MS patients and MSC, SZ patients and SZC were also obtained, respectively. Then, we evaluated the differential microbiota and cytokines that were shared by both diseases. Combined with the associations between microbiota and cytokines, as well as the predicted functions of differential microbiota, the potential roles of gut microbiota in driving the development of both diseases were estimated.

### 2.3. Processing of 16S rRNA Sequences

Read sequences deposited in both datasets were processed with QIIME2 (version 2022.8). Sequence processing procedures such as quality control, clustering of amplicon sequence variants (ASVs) were performed with default parameters [21]. The ASV abundance table was constructed based on downsized sampling reads. Taxonomy of ASVs was annotated based on the SILVA database (version 138.1) [22], while functions of them were predicted with Tax4Fun2 [23, 24]. For alpha diversity, parameters Shannon and Simpson were considered. Potential batch effects in the two datasets were evaluated with PERMANOVA (permutational multivariate analysis of variance) and visualized with principal component analysis (PCA).

### 2.4. Statistical Analysis

The differences in the composition of fecal microbiota at different taxonomic levels were analyzed using the linear discriminant analysis effect size method (LEfSe) [25]. For serum cytokines and clinical indicators, normal distribution was firstly estimated using the Kolmogorov–Smirnov test. Then, the Mann–Whitney *U* test or Student's *t*-test was utilized to evaluate the differences between the groups for serum cytokines meeting or not meeting normal distribution. The final differential serum cytokines were selected based on the *P* value obtained from the Mann–Whitney *U* test or Student's *t*-test combined with fold change (FC). R package “VennDiagram” was utilized to illustrate the overlap of differential cytokines associated with both diseases. The correlation networks between gut microbiota and serum cytokines were evaluated using Pearson correlation analysis. All tests of significance were two-sided, and *P* value less than 0.05 was considered statistically significant. All statistical analysis and graphics were conducted with R software (version 4.0.2) unless stated otherwise.

## 3. Results

### 3.1. Subject Characteristics

For validated SZ patients, MS patients, and corresponding controls, clinical information such as age, gender, BMI, and medical and medication histories was collected from the hospital medical record system. No significant differences in age, gender, BMI, and smoking and drinking histories were observed between SZ patients and corresponding controls (SZC, *P* > 0.05). For MS patients, no patients took antibiotics or Yogurt within 1 month or immunosuppressive medications within 3 months. Although one MS patient showed hyperlipidemia, no significant difference was observed (*P* > 0.05) between MS patients and MSC. Moreover, there were no significant differences between MS patients and MSC in age, gender, and BMI, either. Detailed information can be obtained from our previous research studies [15, 16].

### 3.2. MS and SZ Patients Demonstrated Similar Microbial Diversity

After obtaining the relative abundances of ASVs for all fecal samples obtained from controls, MZ, and SZ patients, we first evaluated whether there was any batch effect in microbial datasets since the reads for MS patients and MSC, SZ patients and SZC were not sequenced in a single batch. As shown Figure 1(b), the score plot of principal component analysis (PCA) based on ASV abundance tables, no apparent difference was observed between two datasets (MS and MSC versus SZ and SZC). PERMANOVA analyses further confirmed no batch-to-batch variation (MS and MSC versus SZ and SZC, *P* > 0.05), as well as similar *β*-diversity between MS and SZ patients (*P* > 0.05). Then, we investigated the microbial diversity in each group of samples (MS and MSC, SZ and SZC) using Shannon and Simpson indices (Figures 1(c) and 1(d)). It was revealed that two populations of controls (MSC and SZC) demonstrated similar values of microbial diversity (*P* > 0.05). Moreover, we compared the microbial diversity indices between MS and SZ patients, and it also showed no apparent difference with regard to both indices (*P* > 0.05).

### 3.3. Microbial Features Common in MS and SZ Patients

**Figure 2:**
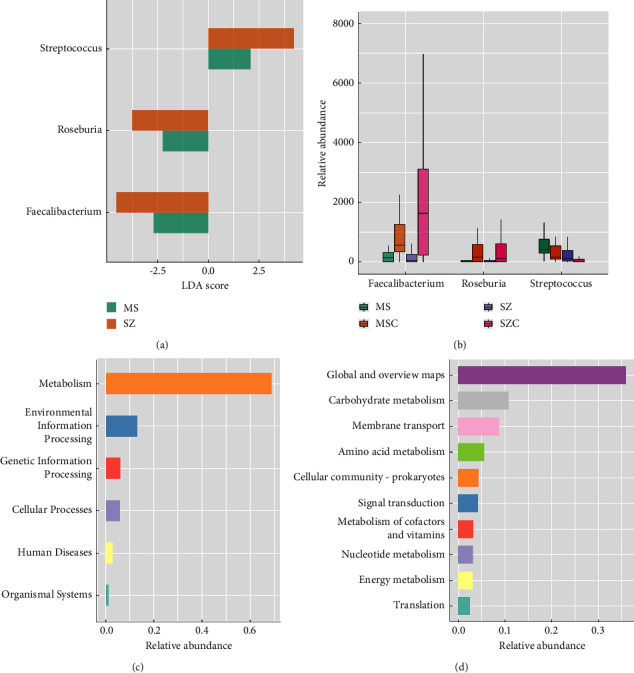
(a) Boxplot illustrating the relative abundances of the three common genera significantly perturbed in MS and SZ patients. (b) LDA scores of the three common genera in datasets MS and SZ. (c, d) Functional pathways associated with the three common genera in different levels.

The microbial features common in MS and SZ patients were conducted using the following two steps. Firstly, to preclude the potential impact of confounding factors such as sex and age, microbiota associated with MS and SZ patients were evaluated by comparing MS, MSC, and SZ, SZC, respectively. Then, the microbial features associated with both kinds of diseases were retrieved by comparing both lists of differential microbiota. Based on *P* value less than 0.05 and LDA score (linear discriminant analysis) higher than 2, three genera were found to be significantly associated with MS and SZ, simultaneously.  Figure 2(a) illustrates the relative abundances of the three genera that were significantly perturbed in MS and SZ patients, while the LDA scores of them are shown in Figure 2(b). It was revealed that two genera *Faecalibacterium* and *Roseburia* decreased in MS or SZ patients as compared to corresponding controls, while *Streptococcus* increased in patients with both kinds of diseases.

We further retrieved the ASVs belonging to the three common genera and predicted the functions of them using Tax4Fun2 [23, 24]. In Figure 2(c), we can see that the most abundant function associated with the three genera was “metabolism,” followed by “environmental information processing,” “genetic information processing,” and “cellular processes.” Among the pathways associated with “metabolism,” “carbohydrate metabolism,” “amino acid metabolism,” “metabolism of cofactors and vitamins,” “nucleotide metabolism,” and “energy metabolism” were the most abundant (Figure 2(d)). Moreover, pathways such as “signal transduction,” “membrane transport,” and “translation” were also found to be associated with the three genera.

### 3.4. Serum Cytokines Associated with Both Kinds of Diseases

**Figure 3:**
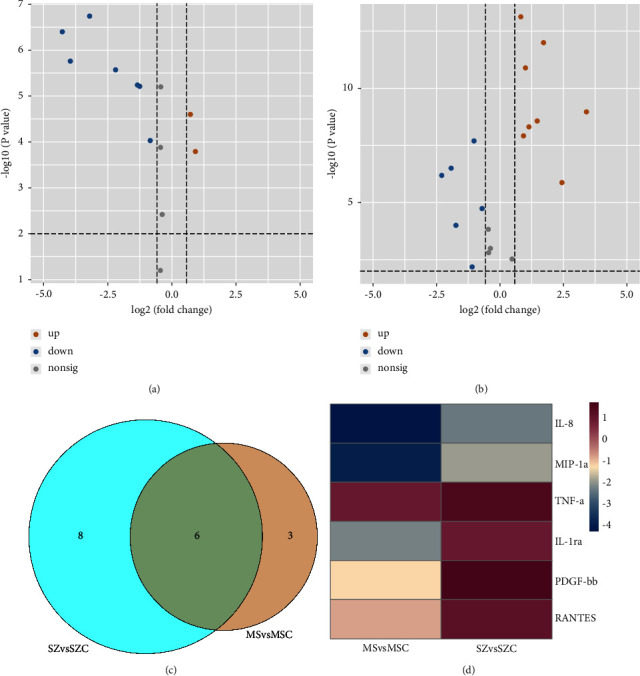
(a) Volcano plot showing the differential serum cytokines between MS patients and corresponding controls. (b) Volcano plot showing the differential serum cytokines between SZ patients and corresponding controls. (c) Venn diagram illustrating the differential serum cytokines shared by both diseases and unique in each disease. (d) Heatmap illustrating the log2 (fold change) values for the six common cytokines in MS and SZ patients versus corresponding controls.

**Figure 4:**
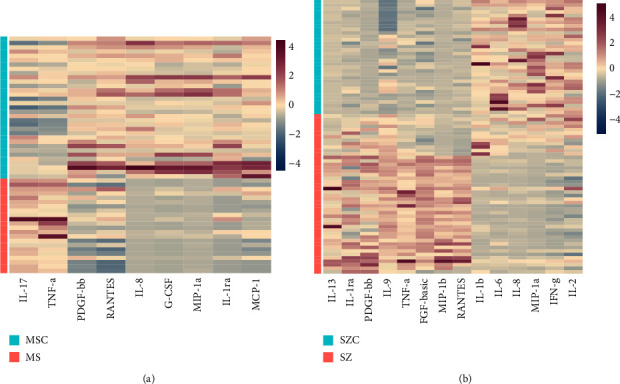
(a) Heatmap illustrating the relative abundances of serum cytokines perturbed in MS patients. (b) Heatmap showing the relative abundances of serum cytokines perturbed in SZ patients.

We also evaluated whether MS and SZ patients shared some patterns of serum cytokines. By retrieving cytokines with *P* values less than 0.01 and fold changes (FC) larger than 1.5, the cytokines significantly differential between MS patients and MSC, SZ patients and SZC were demonstrated in Figures 3(a) and 3(b), with the relative abundances provided in Figures 4(a) and 4(b). The Venn diagram (Figure 3(c)) illustrated that six cytokines were perturbed in both kinds of diseases, while three and eight cytokines were dysregulated specifically in MS and SZ patients as compared to corresponding controls. The log2 (fold change) values for the six cytokines in MS patients versus MSC (MSvsMSC) and SZ patients versus SZC (SZvsSZC) were also evaluated. As shown in Figure 3(d), IL-8 (interleukin 8) and MIP-1a (macrophage inflammatory protein 1-alpha) were downregulated, while TNF-*α* (tumor necrosis factor-alpha) was upregulated in both kinds of disease. Moreover, IL-1ra (interleukin-1 receptor antagonist), PDGF-bb (platelet-derived growth factor BB), and RANTES (regulated upon activation, normal T-cell expressed and presumably secreted) increased in SZ patients but decreased in MS patients as compared to corresponding controls. Such results indicated that although immune responses were present in both kinds of diseases, the patterns of inflammatory responses and underlying mechanisms may not be the same for MS and SZ patients.

### 3.5. Association Networks between Three Genera and Differential Cytokines

**Figure 5:**
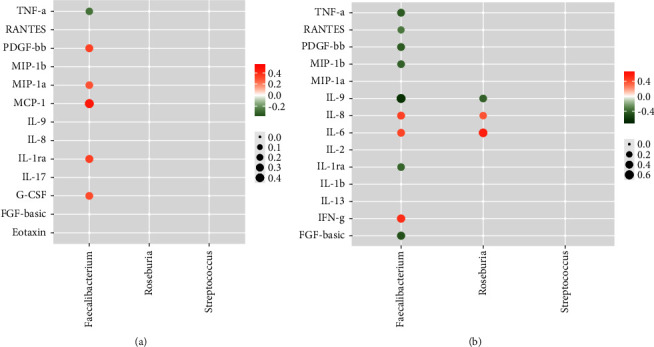
(a) The association network between three common genera and serum cytokines perturbed in MS patients. (b) The association network between three common genera and serum cytokines perturbed in SZ patients.

We further evaluated the associations among three common genera (*Faecalibacterium*, *Roseburia*, and *Streptococcus*) and differential cytokines in each kind of disease. Figures 5(a) and 5(b) demonstrates the association networks for MS and SZ patients, respectively. It was revealed that only *Faecalibacterium* demonstrated significant associations with serum cytokines in MS patients, whereas *Faecalibacterium* and *Roseburia* showed extensive correlations with serum cytokines in SZ patients. For MS patients, *Faecalibacterium* demonstrated positive correlations with PDGF-bb (platelet-derived growth factor BB), MIP-1a, MCP-1 (monocyte chemotactic protein-1), IL-1ra (interleukin-1 receptor antagonist), G-CSF (granulocyte colony stimulating factor), and negative correlation with TNF-*α* (tumor necrosis factor-alpha). For SZ patients, *Faecalibacterium* correlated positively with IL-8 (interleukin-8), IL-6 (interleukin-6), and IFN-*γ* (interferon-gamma) but negatively with TNF-*α*, RANTES, PDGF-bb, MIP-1b (macrophage inflammatory protein 1-beta), IL-9 (interleukin-9), IL-1ra, and FGF-basic (fibroblast growth factor-basic). Other than *Faecalibacterium*, *Roseburia* correlated positively with IL-8 and IL-6 and negatively with IL-9 in SZ patients. In general, *Faecalibacterium* demonstrated the most complicated associations with serum cytokines in both kinds of diseases.

## 4. Discussion

The coexistence of symptoms associated with MS and SZ patients indicated that these two disorders may have similar etiologies. To reveal the mechanisms underlying the coexistence of both diseases, we retrieved and reanalyzed the 16S rRNA sequencing data and serum cytokines from our previous studies. It was revealed that the genera *Faecalibacterium*, *Roseburia*, and *Streptococcus* demonstrated similar trends of perturbation in both diseases, while the patterns of serum cytokines were a bit different. We found that Th17-related responses contributed to MS pathogenesis, as evidenced by the increase of IL-17 in MS patients [26]. Whereas, the inflammatory disorders in SZ patients may be associated with the increased expression of RANTES and MIP-1b since RANTES was thought to promote leukocyte infiltration to sites of inflammation and induce the activation of T-cells, followed by diverse effects including T-cell proliferation or apoptosis and the release of proinflammatory cytokines such as MIP-1b [27, 28]. Moreover, association analysis showed that *Faecalibacterium* correlated extensively with serum cytokines in both diseases.

Inflammation in the periphery and the CNS, reported to be connected to the pathogenesis of MS and SZ [29, 30], was found to be increased in this study, as evidenced by the elevated levels of inflammatory markers such as TNF-*α* in both kinds of diseases. On one hand, TNF-*α* was reported to mediate monocyte infiltration into the intestinal tissues, resulting in tissue damage, disruption of epithelial barrier, and finally leaky gut associated with MS and SZ patients [31, 32]. Bacterial translocation due to the increased intestinal permeability further drove proinflammatory responses in both diseases [33, 34]. Moreover, TNF-*α* was found to stimulate the hypothalamic-pituitary-adrenal (HPA) axis and increase microglial activation in the central nervous system (CNS), which would further contribute to inflammatory responses. The inflammation mediators may further influence the levels of neurotransmitters such as dopamine, noradrenaline, and serotonin and finally induce neuronal damage [35, 36]. In a word, inflammation was an important mediator in the pathogenesis of neurological disorder that may contribute to the coexistence for MS and SZ.

We found in our study that the abundance of *Faecalibacterium* negatively correlated with TNF-*α* and was significantly reduced in both diseases as compared to corresponding controls. *Faecalibacterium* is a kind of Gram-positive bacteria showing anti-inflammatory properties [37]. It was found that *Faecalibacterium* and its supernatant can suppress the expression of TNF-*α* and then alleviate the inflammation in MS [15]. *Faecalibacterium* also produces short chain fatty acids (SCFAs), especially butyrate. Previous studies indicated that butyrate can cross the blood-brain barrier, help improve gut integrity, shape the immune milieu by attenuating proinflammatory cytokine expression in microglia and peripheral, and have a crucial impact on brain plasticity by stimulating the production of BDNF (brain derived neurotrophic factor) in the CNS, which is involved in the survival of neurons [38, 39]. Lower abundance of *Faecalibacterium* may lower the production of butyrate in MS and SZ patients [40], which in turn could contribute to lower BDNF levels, and finally the development of both diseases [41, 42]. Besides *Faecalibacterium*, *Roseburia* was also downregulated in both diseases. Evidences have shown that *Roseburia* played an important role in maintaining gut health by improving the gut ecosystem and exhibiting anti-inflammatory effects by upregulating genes involved in the innate immune responses such as antimicrobial peptides and Toll-like receptors [43, 44]. A reduction in *Roseburia* spp. has been found to contribute to an inflammatory milieu [45]. As another important butyrate-producing bacterium, the reduction of *Roseburia* would further decrease the butyrate level and exacerbate dysfunctions such as gut barrier impairment, peripheral, and brain neuroinflammation associated with the pathophysiology of both diseases.

On the other hand, *Streptococcus* was found to increase in both diseases. Some *Streptococcus* spp. strains were potential pathogens associated with inflammation and an increased risk of a broad spectrum of neuropsychiatric conditions such as SZ [46]. *Streptococcus* spp. strains were also found to be potent acetate and serotonin (5-HT) producers [47]. Increased plasma acetate has been found to correlate with disability and immune response in multiple sclerosis [48]. Although the role of 5-HT in the pathophysiology of SZ and MS is still unclear, it was reported that long-lasting 5-HT overload can disrupt neuronal activity within the cerebral cortex, anterior cingulate cortex, and dorsolateral frontal lobe. The disturbance of the serotonergic system has been found to play an important role in the development of multiple psychiatric disorders ranging from anxiety to SZ [49]. Moreover, a previous study reported that *Streptococcus* spp. can produce neurotoxins such as streptokinase and streptomycin, which might irreversibly damage neurons, including dopaminergic neurons relevant for the pathogenesis of Parkinson's disease [50]. In other words, reduced *Faecalibacterium*, *Roseburia*, and increased *Streptococcus* may together contribute to the brain inflammation and neuronal disorders associated with the coexistence of MS and SZ.

Based on the abovementioned information, the increase of opportunistic pathogen *Streptococcus* and decrease of probiotics (*Faecalibacterium*, *Roseburia*) that played a role in anti-inflammation, improving gut ecosystem and barrier may contribute to the coexistence of MS and SZ via regulating serum cytokines. Supplementing *Faecalibacterium* and *Roseburia* may be helpful in reducing the incidence and co-occurring of both diseases. However, the results of the study were based on statistical analyses, and further studies such as targeted fecal manipulation are still required to confirm the findings of this study.

## 5. Conclusion

In summary, we found that three genera including *Faecalibacterium*, *Roseburia*, and *Streptococcus* were perturbed in a similar way in MS and SZ patients, while the associations of them with serum cytokines were a bit different. MS pathogenesis was found to be associated with Th17-related responses, while the increased expression of RANTES and MIP-1b may contribute to the inflammatory disorders in SZ. Despite the abovementioned discrepancies, increased expression of TNF-*α* indicated the presence of inflammation in both diseases. The reduced *Faecalibacterium* and *Roseburia* and increased *Streptococcus* in together may contribute to systematic inflammation and neuron damages, while *Streptococcus* might also be associated with the dysregulation of neurotransmitters, another possible driver for the neuronal dysfunction. Although further studies with higher depths and resolutions are still required for fully understanding the pathologies relating to both diseases, our study depicted the potential roles of gut microbiome and paved the way for developing gut microbiota-targeted therapies that are capable of managing two diseases with a single treat.

## Data Availability

The datasets generated and/or analyzed during the current study are available in the GenBank Sequence Read Archive with the accession numbers PRJNA807473 (https://www.ncbi.nlm.nih.gov/sra/?term=PRJNA807473) and SRP258890 (PRJNA628832, https://www.ncbi.nlm.nih.gov/sra/?term=PRJNA628832).
